# Construction and Validation of an Immune-Based Prognostic Model for Pancreatic Adenocarcinoma Based on Public Databases

**DOI:** 10.3389/fgene.2021.702102

**Published:** 2021-07-14

**Authors:** Miaobin Mao, Hongjian Ling, Yuping Lin, Yanling Chen, Benhua Xu, Rong Zheng

**Affiliations:** ^1^The Graduate School, Fujian Medical University, Fuzhou, China; ^2^Department of Radiation Oncology, Fujian Medical University Union Hospital, Fuzhou, China; ^3^Union Clinical Medicine College, Fujian Medical University, Fuzhou, China; ^4^Department of Hepatobiliary Surgery, Fujian Medical University Union Hospital, Fuzhou, China; ^5^College of Medical Technology and Engineering, Fujian Medical University, Fuzhou, China; ^6^School of Clinical Medicine, Fujian Medical University, Fuzhou, China

**Keywords:** pancreatic adenocarcinoma, immune-related genes, transcription factors, prognostic model, tumor immune microenvironment

## Abstract

**Background:**

Pancreatic adenocarcinoma (PAAD) is a highly lethal and aggressive tumor with poor prognoses. The predictive capability of immune-related genes (IRGs) in PAAD has yet to be explored. We aimed to explore prognostic-related immune genes and develop a prediction model for indicating prognosis in PAAD.

**Methods:**

The messenger (m)RNA expression profiles acquired from public databases were comprehensively integrated and differentially expressed genes were identified. Univariate analysis was utilized to identify IRGs that related to overall survival. Whereafter, a multigene signature in the Cancer Genome Atlas cohort was established based on the least absolute shrinkage and selection operator (LASSO) Cox regression analysis. Moreover, a transcription factors regulatory network was constructed to reveal potential molecular processes in PAAD. PAAD datasets downloaded from the Gene Expression Omnibus database were applied for the validations. Finally, correlation analysis between the prognostic model and immunocyte infiltration was investigated.

**Results:**

Totally, 446 differentially expressed immune-related genes were screened in PAAD tissues and normal tissues, of which 43 IRGs were significantly related to the overall survival of PAAD patients. An immune-based prognostic model was developed, which contained eight IRGs. Univariate and multivariate Cox regression revealed that the risk score model was an independent prognostic indicator in PAAD (HR > 1, *P* < 0.001). Besides, the sensitivity of the model was evaluated by the receiver operating characteristic curve analysis. Finally, immunocyte infiltration analysis revealed that the eight-gene signature possibly played a pivotal role in the status of the PAAD immune microenvironment.

**Conclusion:**

A novel prognostic model based on immune genes may serve to characterize the immune microenvironment and provide a basis for PAAD immunotherapy.

## Introduction

Pancreatic cancer (PC) is one of the deadliest and most aggressive malignant neoplasms worldwide (Ilic and Ilic, 2016). In the next decade, PC is estimated to be the second leading cause of death among malignant cancer-related diseases (Rahib et al., 2014; Ferlay et al., 2016). Pancreatic adenocarcinoma (PAAD) occurs in approximately 85% of all PC cases and is associated with a less favorable prognosis (Higuera et al., 2016).

Pancreatic cancer treatment comprises surgery, chemotherapy, radiotherapy, neoadjuvant therapy, targeted molecular therapy, and immunotherapy. Nevertheless, the therapeutic effect of these strategies for PAAD is limited. Therefore, accurate prediction of the prognosis can determine if the patient will benefit from more radical treatment, thereby providing the patient with “individualized” systemic treatment to improve the prognosis.

Pancreatic adenocarcinoma is characterized by the high complexity of stomal tissue, which includes immune cells, various growth factors, the extracellular matrix, and fibroblasts. The tumor microenvironment (TME) accounts for about 15–85% of the whole tumor component in PAAD (Erkan et al., 2012; Liang et al., 2017). The complex and heterogeneous TME induced by interactions between pancreatic epithelial/cancer cells and stromal cells is responsible for PC progression and has been implicated in resistance to chemotherapy and immunotherapy (Markowitz et al., 2015; Incio et al., 2016; Ren et al., 2018). Besides, components of the PAAD microenvironment that contribute to immunosuppression correlate with a poor prognosis of patients (Tang et al., 2014; Whatcott et al., 2015; Wang et al., 2017). With deepening of the understanding of the microenvironment of PC, TME-based clinical and translational therapies could be a breakthrough hotspot in PC treatment in the future.

With the remarkable progress of bioinformatics analysis, in many studies, the mining of public databases has been used increasingly to predict cancer prognosis. Among them, immune-related genes (IRGs) have shown an increasingly prominent role in cancer development and immunotherapy (Ge et al., 2019; Huang et al., 2020; Kong et al., 2020; Yang et al., 2020). Predictive biomarkers related to the tumor immune microenvironment are expected to identify additional target molecules and to enhance immunotherapy efficacy (Taube et al., 2018; Bianco et al., 2019; Jiang et al., 2019; Liu et al., 2020; Zhao B. et al., 2020; Zwing et al., 2020). Currently, PC still lacks prognostic biomarkers related to the tumor immune microenvironment. Therefore, it is necessary to explore important biomarkers in PAAD to guide appropriate treatment options to improve the therapeutic efficacy of patients.

In our research, we investigated the messenger (m)RNA expression and corresponding clinical information of PAAD patients from public databases. Next, we constructed an IRGs-based prognostic model in The Cancer Genome Atlas (TCGA) cohort and validated it in the Gene Expression Omnibus (GEO) dataset. The regulatory network structured by differentially expressed transcription factors (DETFs) and prognosis-related IRGs may provide a theoretical basis to reveal the potential mechanisms at the molecular level. Finally, analyses of prognostic “gene signatures” and infiltration of immune cells may provide new ideas for the role of IRGs in predicting PAAD prognosis.

## Materials and Methods

### Data Acquisition

The transcriptome sequencing data and corresponding clinical data of 176 PAAD patients were extracted from TCGA (172 PAAD specimens and four normal tissue specimens). The RNA-sequencing data of normal pancreatic tissue were acquired from the Genotype-Tissue Expression (GTEx) Project^1^ as well (Carithers et al., 2015). It contains the RNA-expression profile of 167 normal pancreatic tissues. Meanwhile, RNA sequencing fragments per kilobase of exon model per million reads mapped (FPKM) data were also obtained for further analyses. For validation cohort, gene expression matrix files and clinical data of 125 patients with PAAD in the GSE71729 dataset were downloaded from the GEO^2^. Match the gene symbols corresponding to the probes according to the annotation file provided by the manufacturer. If a single gene matches multiple probes, the median ranking value accounts for the expression value. We normalized gene expression value using the robust multiarray average (RMA) algorithm, and the normalized data were log2-transformed for further analyses. Publishing guidelines provided by the GEO database were observed, Therefore, there was no requirement for additional ethical approval. Furthermore, a list of IRGs was acquired from the Immunology Database and Analysis Portal (ImmPort) database that shares resources for immunology-related research^3^ (Bhattacharya et al., 2014). Then, a list of transcription factors (TFs) was obtained from the Cistrome Project^4^, including 318 TFs (Mei et al., 2017).

### Analyses of Differentially Expressed Genes in PAAD

The “limma” R package^5^ (Ritchie et al., 2015) was used for analyses of differential expression. Differential gene expression was defined with adjusted-*P* < 0.01 and | log2 fold change| > 2 as the cutoff criteria. Then, we extracted differentially expressed immune-related genes (DEIRGs) and DETFs from all DEGs based on the lists obtained from ImmPort and Cistrome Cancer databases.

### Analyses of DEIRGs in PAAD Using the Gene Ontology and Kyoto Encyclopedia of Genes and Genomes Databases

The functions and pathway enrichment of candidate DEIRGs were analyzed using Database for Annotation, Visualization and Integrated Discovery (DAVID) v6.8^6^ (Dennis et al., 2003). To explore the underlying biological functions of DEIRGs, the GO and KEGG databases were searched using the R packages “GOplot^7^” (Walter et al., 2015) and “clusterProfiler^8^” (Kanehisa et al., 2017), respectively. Moreover, the cutoff value for pathway screening and significant functionality was placed at *P* < 0.05. To explain the correlation between enriched pathways and prognostic IRGs, an interaction network was constructed for visual representations.

### Transcription Factors-Mediated Prognosis-Related IRGs Modulation Network

A short duration of follow-up usually limits the accuracy of survival analyses. Hence, we selected patients whose duration of follow-up was ≥60 days. To investigate the prognosis-related DEIRGs in PAAD patients, the “survival” R package^9^ was applied to implement the univariate Cox regression analysis (*P* < 0.01). To explore the interactions between DETFs and prognosis-related DEIRGs, the correlation test function was employed (set thresholds: *P* < 0.001 and correlation coefficient > 0.4).

### Construction of the IRGs-Based Prognostic Model for PAAD

An IRG prognostic model was developed based on the LASSO Cox regression analysis. To minimize the risk of overfitting, the Lasso method used 10-fold cross validation based on the “glmnet” package^10^ in R (R Project for Statistical Computing, Vienna, Austria) (Tibshirani, 1997; Simon et al., 2011). Then, we used β coefficients of the LASSO Cox regression analysis to establish the DEIRGs-based prognostic model for PAAD. We used it to establish a formula to predict the risk score of each patient. The receiver operating characteristic (ROC) curve was used to judge the discrimination ability of various statistical methods on the basis of the binary gold standard (Hanley and McNeil, 1982). The ROC curve was created by the “survival ROC” R package^11^ to evaluate the sensitivity of the model. Finally, principal component analysis (PCA) was done based on the “prcomp” function from the “stats” R library.

### Correlation Between the Immune-Related Signature and Clinical Features in a Prognostic Model of PAAD

The relevance between clinical characteristics (age, gender, histology grade, tumor stage, T staging, N staging, M staging, residual tumor, and outcomes) and expression of eight prognosis signatures in the prognostic model was analyzed using the “beeswarm” R package.

### Further Verification of a Prognostic IRG Signature

To verify the prognostic value of the immune-related signature risk score model, we used the GSE71729 dataset as the validation cohort. Samples in the GSE71729 cohort were then divided into high-risk and low-risk groups based on the optimal cut-off point. Kaplan–Meier and ROC curve analysis of the eight-gene signature were performed as mentioned above. In addition, the Human Protein Atlas^12^ (Pontén et al., 2011) was used to extract the protein expression of prognostic-related immune genes in tumor samples and normal samples.

### Analysis of Immunocyte Infiltration

The Tumor Immune Estimation Resource (TIMER) was employed to analyze and visualize the abundance of tumor-infiltrating immunocytes^13^ (Li et al., 2017). It detailed the abundance of six subsets of tumor-infiltrating immunocytes: B cells, CD8^+^T cells, CD4^+^T cells, macrophages, neutrophils, and dendritic cells (DCs). The online “Immune Estimation” file was retrieved, and the potential correlation between the prediction model and tumor-infiltrating immunocytes was conducted in R.

### Statistical Analysis

Statistical analysis was undertaken with R v3.6.3. Unless specified otherwise, *P* < 0.05 was considered significant.

## Results

**FIGURE 1:**
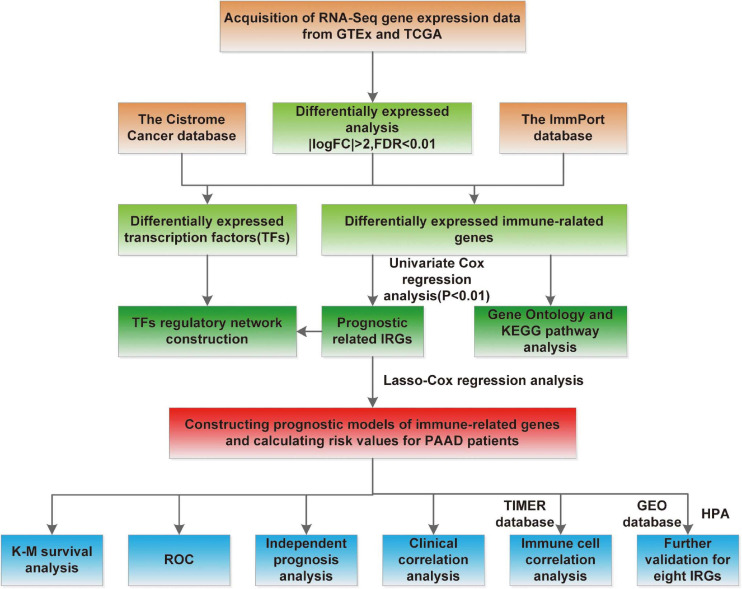
Study flowchart.

The flowchart of our study was displayed in Figure 1. The clinical features of the 185 PAAD patients enrolled in the TCGA–PAAD cohort were presented in Table 1.

**TABLE 1:** Clinical features of patients with pancreatic adenocarcinoma (PAAD).

Clinical characteristics	Patients (*n* = 185)	Percentage (%)
**Age (years)**		
≤65	96	51.9
>65	89	48.1
**Sex**		
Female	83	44.9
Male	102	55.1
**Grade**		
G1	32	17.3
G2	97	52.4
G3	51	27.6
G4	2	1.1
GX	3	1.6
**Stage**		
I	21	11.4
II	152	82.2
III	4	2.2
IV	5	2.7
NA	3	1.6
**Residual tumor**		
R0	111	60.0
R1	53	28.6
R2	5	2.7
NA	16	8.6
**Outcome**		
CR^1^	70	37.8
PR^2^	9	4.9
SD^3^	9	4.9
PD^4^	57	30.8
NA	40	21.6

*^1^CR, complete response; ^2^PR, partial response; ^3^SD, stable disease; ^4^PD, progressive disease.*

### Identification of DEIRGs and TFs in PAAD

**FIGURE 2:**
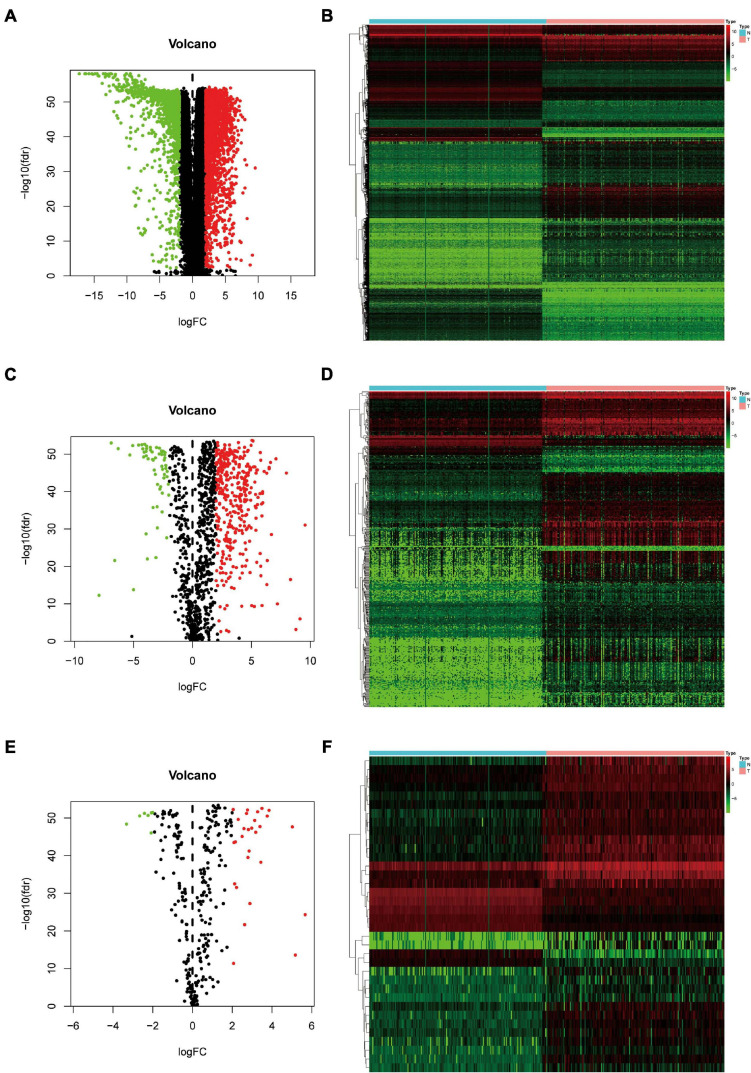
Identification of differentially expressed genes (DEGs), immune-related genes (IRGs), and transcription factors (TFs) in Pancreatic adenocarcinoma (PAAD) *vs*. normal tissues. **(A)** Volcano plot revealing clusters of DEGs with upregulated and downregulated expression. **(B)** The distinction between DEG expression in tumor tissues and normal tissues revealed by a hierarchical clustering heatmap. **(C)** Volcano plot demonstrates clusters of differentially expressed immune-related genes (DEIRGs) with upregulated and downregulated expression. **(D)** Heatmap showing the distinction between expression of DEIRGs in tumor tissues and normal tissues. **(E)** Volcano plot showing clusters of differentially expressed transcription factors (DETFs) with upregulated and downregulated expression. **(F)** Discrimination between DETFs expression in tumor tissues and normal tissues revealed by a heatmap.

A total of 343 tissues were analyzed [172 PAAD tissues and 171 normal tissues (167 from the GTEx database)]. Compared with normal tissue specimens, 4,194 genes (expression of 2,313 was upregulated and expression of 1,881 was downregulated; Supplementary Table 1), 446 IRGs (expression of 387 was upregulated and expression of 59 was downregulated; Supplementary Table 2) and 36 TFs (expression of 29 was upregulated and expression of seven was downregulated; Table 2) were identified as differentially expressed in PAAD tissues (set threshold: *P* < 0.01, fold change > 2). The results mentioned above were shown as a heatmap and volcano map (Figure 2).

**TABLE 2:** Differentially expressed transcription factors (TFs).

TF	Non-tumor (mean)	Tumor (mean)	Log FC^1^	*P* value	FDR^2^
HOXB7	0.624741	7.039385	3.494119	7.91 × 10^–55^	2.71 × 10^–53^
LMNB1	1.356783	5.614074	2.048858	2.02 × 10^–54^	5.34 × 10^–53^
E2F1	0.47699	3.251045	2.768873	2.93 × 10^–54^	7.12 × 10^–53^
BATF	0.696809	10.0601	3.851738	3.84 × 10^–54^	8.76 × 10^–53^
FOXP3	0.154277	1.493511	3.275111	1.25 × 10^–53^	2.13 × 10^–52^
SMAD2	6.780617	1.690707	−2.00379	2.19 × 10^–53^	3.27 × 10^–52^
MEIS1	14.091	3.360955	−2.06783	2.96 × 10^–53^	4.07 × 10^–52^
BRF1	6.292508	1.1787	−2.41644	4.50 × 10^–53^	5.60 × 10^–52^
VDR	0.980843	6.460891	2.719638	4.74 × 10^–53^	5.80 × 10^–52^
SIX5	9.490325	2.016525	−2.23459	2.24 × 10^–52^	1.97 × 10^–51^
GTF2I	18.92405	3.002807	−2.65584	3.28 × 10^–52^	2.72 × 10^–51^
GATA3	0.180424	2.441417	3.758254	3.72 × 10^–52^	3.02 × 10^–51^
PRDM1	0.660779	3.259162	2.302262	3.62 × 10^–51^	2.26 × 10^–50^
EGR2	0.578491	5.060113	3.128803	7.21 × 10^–51^	4.26 × 10^–50^
EPO	2.29224	0.230069	−3.31662	8.16 × 10^–50^	4.07 × 10^–49^
FOSL1	0.866588	9.11024	3.394071	4.07 × 10^–49^	1.84 × 10^–48^
SPDEF	0.307047	9.991052	5.024106	4.98 × 10^–49^	2.23 × 10^–48^
CENPA	0.182789	1.443159	2.98098	1.09 × 10^–48^	4.68 × 10^–48^
LEF1	0.609301	3.669304	2.590279	1.50 × 10^–48^	6.35 × 10^–48^
MYB	0.105902	0.743503	2.811615	2.54 × 10^–48^	1.05 × 10^–47^
NCAPG	0.208888	1.467141	2.812209	2.88 × 10^–48^	1.19 × 10^–47^
KLF5	3.126404	28.38247	3.182424	1.52 × 10^–47^	5.88 × 10^–47^
FOXP2	1.660682	0.392566	−2.08077	2.35 × 10^–47^	8.93 × 10^–47^
PPARG	1.176273	6.645312	2.498113	2.19E-46	7.75 × 10^–46^
BHLHE40	14.81576	66.10077	2.157535	6.77 × 10^–45^	2.15 × 10^–44^
FOXM1	0.749049	3.172979	2.082705	9.94 × 10^–45^	3.13 × 10^–44^
TFAP2A	0.241558	1.740172	2.848786	2.09 × 10^–42^	5.82 × 10^–42^
FOXA1	0.314465	2.174509	2.789721	1.14 × 10^–40^	2.95 × 10^–40^
HOXC9	0.104638	1.134456	3.438516	2.26 × 10^–39^	5.55 × 10^–39^
E2F7	0.12603	0.548239	2.121042	1.47 × 10^–33^	3.02 × 10^–33^
IRF4	0.180677	0.839005	2.215269	1.41 × 10^–32^	2.81 × 10^–32^
TP63	0.0931	0.689169	2.88801	2.86 × 10^–28^	5.14 × 10^–28^
HOXC11	0.014602	0.74272	5.668628	2.76 × 10^–25^	4.67 × 10^–25^
SOX2	0.106565	0.65707	2.624311	1.33 × 10^–22^	2.13 × 10^–22^
HOXB13	0.016004	0.577841	5.174176	1.80 × 10^–14^	2.47 × 10^–14^
MYH11	3.82786	16.02631	2.065833	3.11 × 10^–12^	4.09 × 10^–12^

*^1^logFC, log fold change (tumor tissues vs. non-tumor tissues); ^2^FDR, false discovery rate.*

### Functional and Pathway Analyses Using GO and KEGG Databases

**FIGURE 3:**
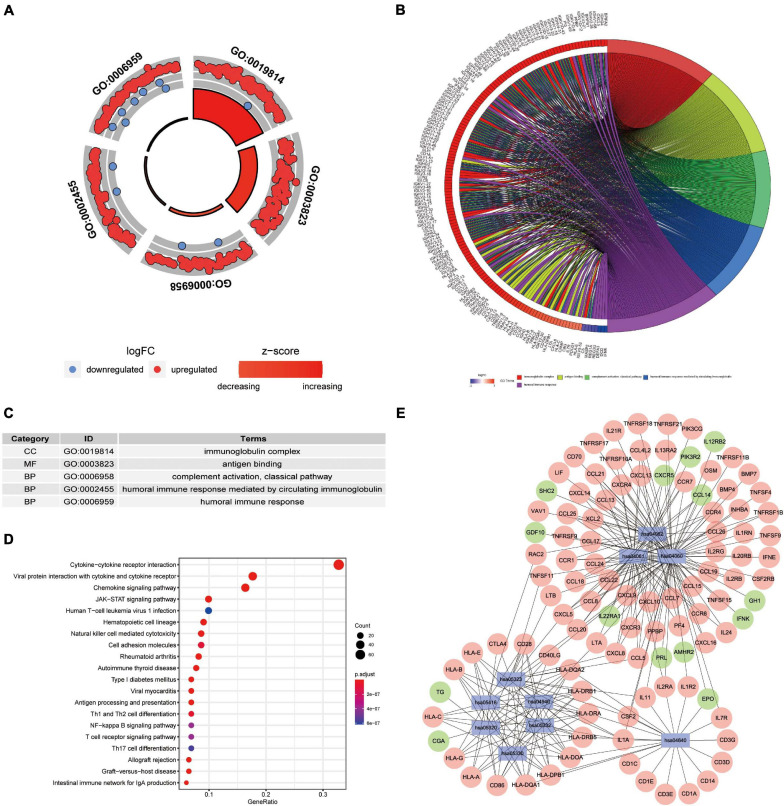
Functional-enrichment analyses of DEIRGs in PAAD. **(A)** The outer circle shows expression (log FC) of DEIRGs in each enriched Gene Ontology (GO) term: red dots which are on each GO term indicate upregulation of DEIRGs. Blue dots indicate downregulated DEIRGs. The inner circle shows the prominence of GO terms (log10-adjusted *P*-values). **(B)** The circle represents the relationship between the top-five most significant GO terms and their related DEIRGs. **(C)** The top-five most significant GO terms and their annotations. **(D)** The top-20 pathways enriched in DEIRGs are shown in the bubble plot. **(E)** The top-10 pathways and the corresponding DEIRGs. The blue rectangles represent Kyoto Encyclopedia of Genes and Genomes (KEGG) pathways. The red ellipses indicate upregulated DEIRGs. The green ellipses indicate downregulated DEIRGs.

We wished to elucidate the biological properties and pathways of DEIRGs in PAAD patients. Hence, the GO and KEGG databases were employed. Inevitably, the DEIRGs were enriched in several immune-related molecular functions. The correlation between the top-five most important GO terms and their related DEIRGs was displayed (adjusted-*P* < 0.05; Figures 3A–C). Among them, “GO: 0019814 immunoglobulin complex” was the most prominent GO term. Figure 3D displays the top-20 significant pathways. The “pathway-DEIRGs” network (Figure 3E) was used for visualizing the reciprocity between the top-10 significant pathways and DEIRGs. Supplementary Table 4 shows 57 significant pathways according to the KEGG database. Adjusted-*P* < 0.05 was considered indicative of significance. Based on visualized data mining, hsa04060 (“cytokine–cytokine receptor interaction”), hsa04061 (“viral protein interaction with cytokine and cytokine receptor”), and hsa04062 (“chemokine signaling pathway”) were used more often to validate our findings using the KEGG database.

### Regulatory Network of TFs

**FIGURE 4:**
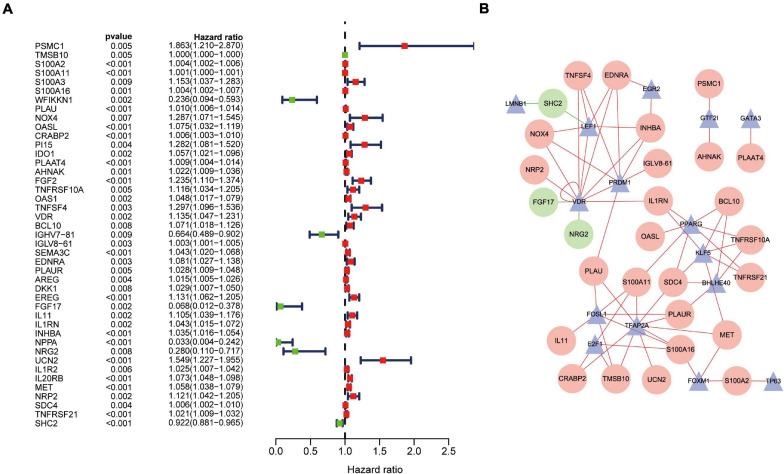
Overall survival (OS)-related DEIRGs and TFs-IRGs regulatory network. **(A)** The forest plot of OS-related DEIRGs in PAAD. Red and green dots indicate high risk and low risk, respectively. **(B)** Regulatory network between prognosis-related DEIRGs and DETFs in PAAD. The red and green circles indicate high-risk and low-risk DEIRGs, respectively. The blue triangles indicate DETFs. The red and green lines represent positive and negative correlation, respectively.

Univariate Cox regression analysis revealed that 43 DEIRGs were associated with overall survival (OS) (*P* < 0.01): 37 high-risk IRGs and six low-risk IRGs (Figure 4A). We constructed a regulatory network based on 54 DEIRGs and 36 DETFs (set threshold: *P* < 0.001; correlation coefficient > 0.4). According to the cutoff criteria, 29 prognostic-related DEIRGs and 14 DETFs (Figure 4B) participated in the establishment of the network. Finally, the regulatory network was constructed and visualized using Cytoscape software^14^. The TFs lamin B1 (LMNB1) and lymphoid enhancer-binding factor 1 (LEF1) act as negative regulators of IRG SHC adaptor protein 2 (SHC2) (Figure 4B). Besides, the TF vitamin-D receptor (VDR) had a negative relationship with IRG fibroblast growth factor 17 (FGF17) and neuregulin 2 (NRG2). The specific regulatory relationship between TFs and OS-related IRGs in PAAD was listed in Supplementary Table 5.

### Construction of an Eight-IRGs Prognostic Model

Least absolute shrinkage and selection operator Cox regression analysis was applied to build a prognostic model based on the expression profile of the 43 prognostic DEIRGs mentioned above. Finally, eight genes were selected to construct a prognostic model based on the optimal value of λ (Supplementary Figure 1). The specific formula for the calculation was:

$$\text{Risk}\,\text{score}={\text{e}}^{\begin{array}{c} {}[\left(-0.1301\right)\times \mathrm{expression}\mathrm{of}\mathrm{WFIKKN1}\\ +0.0016\times \mathrm{expression}\,\mathrm{of}\,\mathrm{PLAU}+0.0004\\ \times \mathrm{expression}\,\mathrm{of}\,\mathrm{OASL}+\left(-0.2278\right)\\ \times \mathrm{expression}\,\mathrm{of}\,\mathrm{FGF17}+\left(-0.3925\right)\\ \times \mathrm{expression}\,\mathrm{of}\,\mathrm{NPPA}+\,0.0258\\ \times \mathrm{expression}\,\mathrm{of}\,\mathrm{IL20}\,\text{RB}+0.0251\\ \times \mathrm{expression}\,\mathrm{of}\,\mathrm{MET}+\left(-0.0081\right)\\ \times \mathrm{expression}\mathrm{of}\mathrm{SHC2}] \end{array}}$$

**FIGURE 5:**
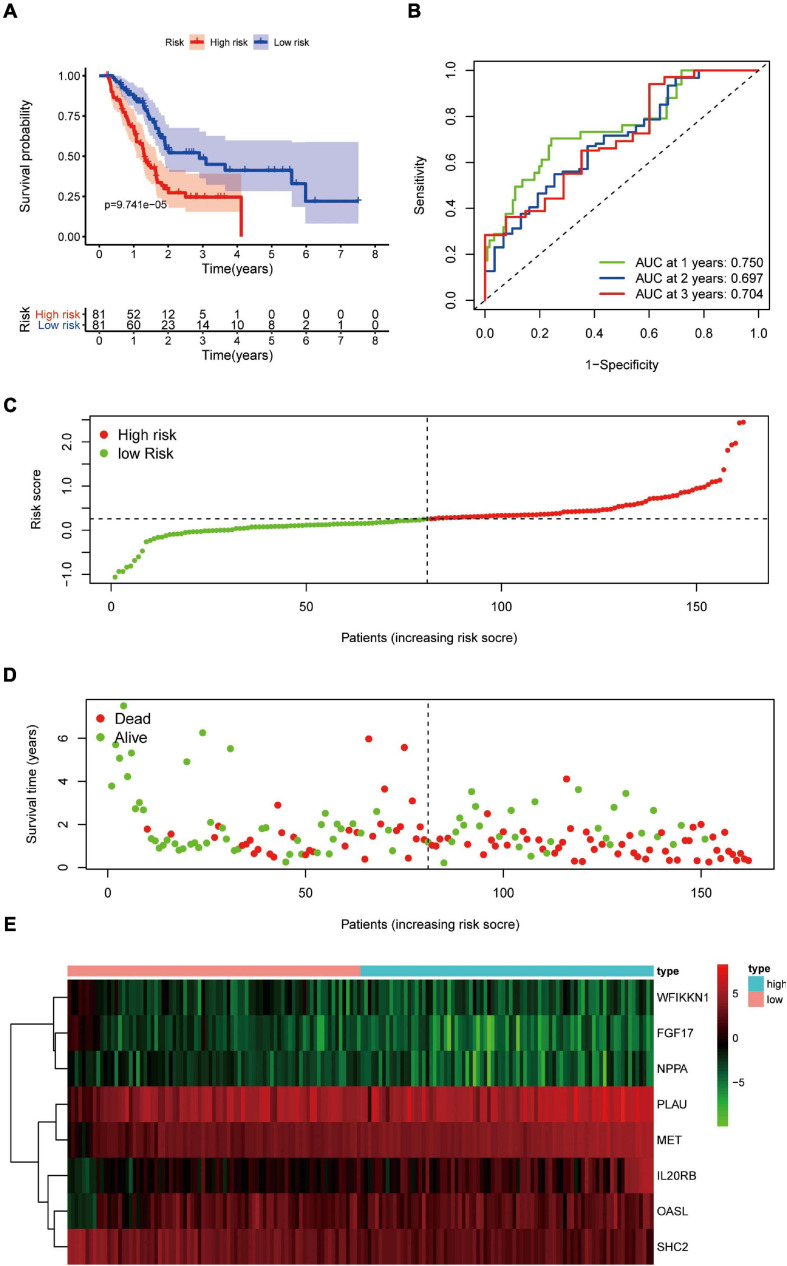
Prognostic value of eight DEIRGs in PAAD patients. **(A)** Analyses of Kaplan–Meier curves for OS in PAAD patients using the signature of eight DEIRGs. **(B)** Receiver operating characteristic (ROC) curve suggesting the feasibility of our prognostic model. **(C)** Patients in high-risk (red dots) and low-risk (green dots) groups and the distribution of their corresponding risk score. **(D)** Patients in high-risk (red dots) and low-risk (green dots) groups, and their corresponding survival status. **(E)** Discrimination of expression of eight prognosis-related IRGs between high-risk and low-risk groups as revealed by a heatmap.

**FIGURE 6:**
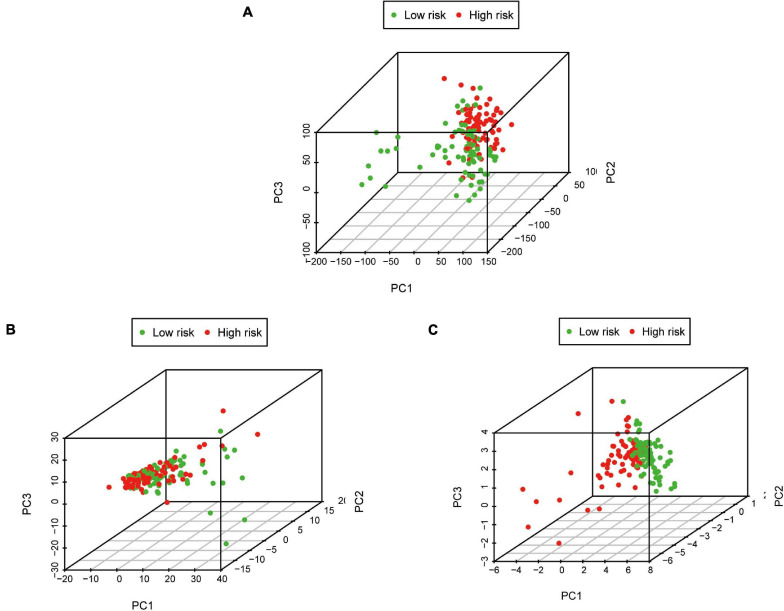
Principal component analysis between low-risk and high-risk groups based on different classification methods. **(A)** All genes, **(B)** Immune genes, and **(C)** Risk genes.

Pancreatic adenocarcinoma patients were separated into a high-risk group (*n* = 81) and a low-risk group (*n* = 81) based on the median value of the risk score (Figure 5C). PCA was undertaken to study the differences between low- and high-risk populations using the expression profiles of all genes, IRGs, and risk-related genes (Figure 6). We discovered that low- and high-risk groups were distributed in different directions (Figure 6C). Patients with high risk were more likely to die sooner than those with a low risk (Figure 5D). The Kaplan–Meier curve demonstrated that patients with high risk showed markedly worse OS than those with low risk (*P* < 0.001; Figure 5A). The area under the time-dependent ROC curves for 1-, 2-, and 3-years OS reached 0.750, 0.697, and 0.707 respectively. Hence, the predictive performance of the prognostic model exhibited good sensitivity and specificity (Figure 5B). Also, the immune-based prognostic model was relatively consistent. Figure 5E shows the expression of eight IRGs in the form of a heatmap.

### Independent Prognostic Value of the Eight-Gene Signature

**FIGURE 7:**
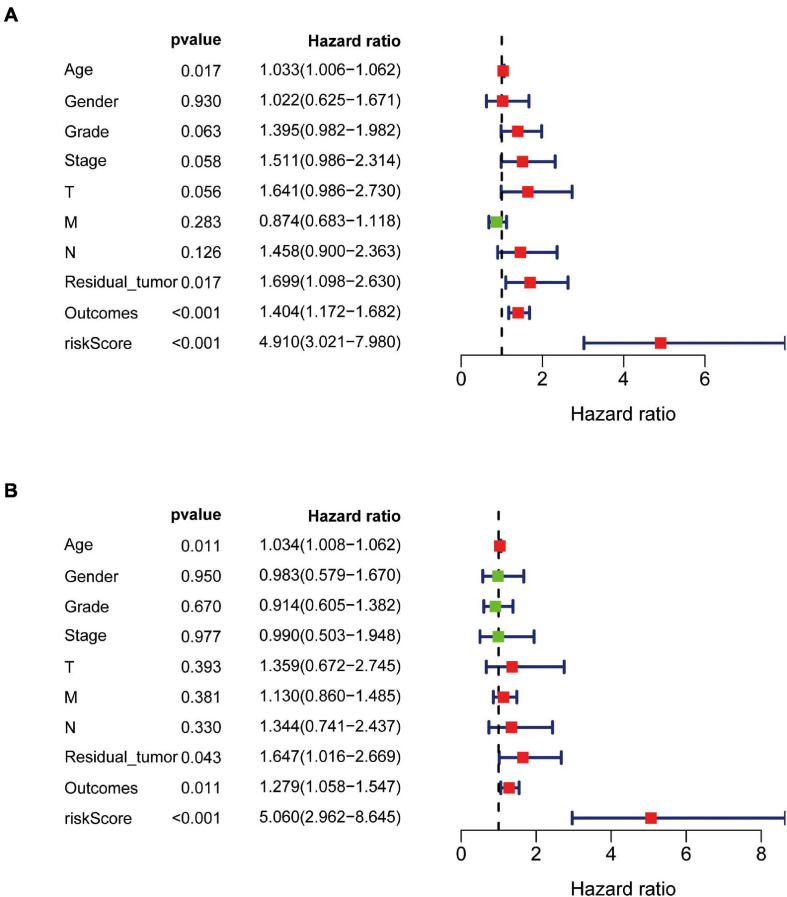
Univariate and multivariate independent prognostic analysis in PAAD. **(A,B)** Forest plots of univariate and multivariate independent prognostic analysis.

The independent predictive value of the prognostic signature was assessed by univariate and multivariate Cox regression analyses. Univariate Cox prognostic analyses demonstrated that the risk score was correlated significantly with OS [hazard ratio (HR) = 4.910, 95% confidence interval (CI) = 3.021–7.980, *P* < 0.001] (Figure 7A). After the multivariate analysis, the risk score remained an independent prognostic factor correlated with OS (HR = 4.868, 95% CI = 2.899–8.175, *P* < 0.001; Figure 7B). Moreover, univariate and multivariate independent prognostic analyses (Figures 7A,B) showed that the residual tumor and outcomes were also significant independent prognostic factors for survival (*P* < 0.05; Table 3).

**TABLE 3:** Univariate and multivariate independent prognostic analyses.

Variable	Univariate analysis		Multivariate analysis	
	HR (95%CI)	*p*	HR (95%CI)	*p*
Age	1.033 (1.006–1.062)	0.017	1.038 (1.012–1.065)	0.005
Sex	1.022 (0.625–1.671)	0.930	1.008 (0.592–1.715)	0.978
Grade	1.395 (0.982–1.982)	0.063	0.908 (0.601–1.371)	0.647
Stage	1.511 (0.986–2.314)	0.058	1.252 (0.788–1.989)	0.341
Residual tumor	1.699 (1.098–2.630)	0.017	1.678 (1.047–2.690)	0.032
Outcomes	1.404 (1.172–1.682)	<0.001	1.244 (1.030–1.503)	0.023
Risk score	4.910 (3.021–7.980)	<0.001	4.868 (2.899–8.175)	<0.001

### Eight-IRG Prognostic Model and Clinical Characteristics

**FIGURE 8:**
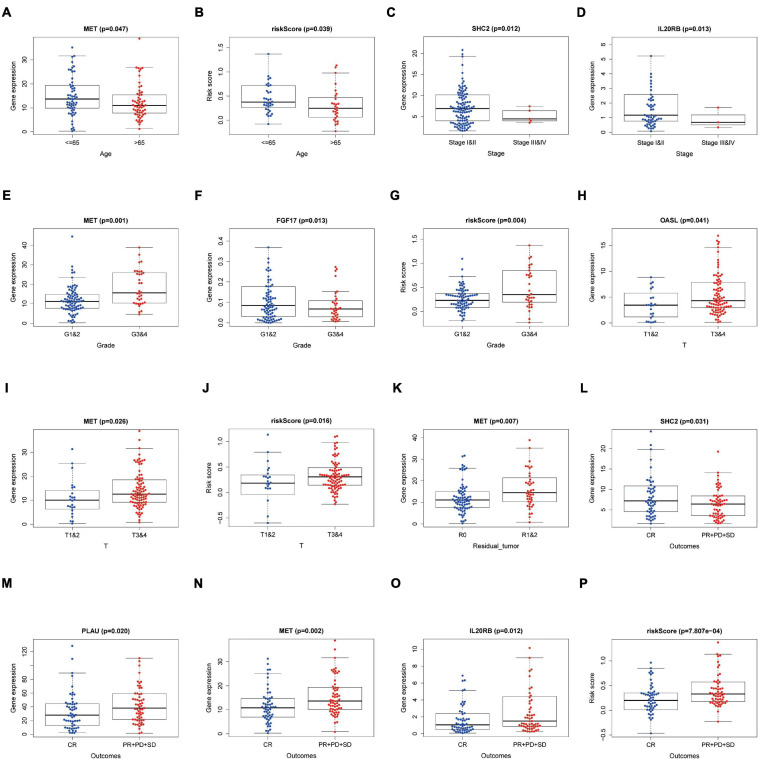
Relationships between the clinical-pathological characteristics and expression of DEIRGs in PAAD. **(A)** Differences in expression of DEIRGs between the pathological TNM stages I and II/III and IV in PAAD. **(B,C)** Differences in expression of DEIRGs between the histology G1 and 2/G3 and 4 grades in PAAD. **(D,E)** Differences in expression of DEIRGs between the T stages T1 and 2/T3 and 4 in PAAD. **(F–K)** Differences in expression of DEIRGs between the M stages M0/M1/MX in PAAD. **(L)** Differences in expression of DEIRGs between the residual tumor R0/R1 and R2 in PAAD. **(M–P)** Differences in expression of DEIRGs between the outcomes (CR/PR + PD + SD) in PAAD.

Relationships between eight IRGs in the risk-score model and clinical features (age, gender, pathological TNM stage, histology grade, residual tumor, and outcomes) were assessed via the beeswarm packages in R (*P* < 0.05; Table 4). The cutoff value was determined by the median of the expression of the selected genes. As observed from Figure 8, the median values in the age ≤ 65 group were higher than those in the age > 65 group between mesenchymal epithelial transition factor (MET) expression and riskscore (Figures 8A,B). The median value of the SHC2 and interleukin 20 receptor subunit beta (IL20RB) expression in pathological stage I-II was higher than that in stage III-IV (Figures 8C,D). With regard to histology grade, the median value of MET expression and riskscore in grades 1 and 2 was lower than that in grade 3 and 4, and the trend was exactly the opposite for Fibroblast Growth Factor (FGF)17 (Figures 8E–G). The median values in T1–2 staging were lower than those in T3–4 staging among 2′–5′-oligoadenylate synthetase like (OASL) expression, MET expression, and riskscore (Figures 8H–J). Moreover, the median value of MET expression was lower in residual tumor R0 than that in R1 and 2 (Figure 8K). Additionally, the median values of SHC2, plasminogen activator, urokinase (PLAU) expression, MET expression, IL20RB expression, and riskscore were notably different in PAAD at outcomes CR relative to those at PR + PD + SD (*P* < 0.05, Figures 8L–P).

**TABLE 4:** Correlation between clinical features.

Gene	Age (≤65/>65)		Sex (male/female)		Grade (G1 and 2/G3 and 4)		Stage (I and II/III and IV)		Residual tumor (R0/R1 and 2)		Outcome (CR/PR and PD and SD)	
	*t*	*P*	*t*	*P*	*t*	*P*	*t*	*P*	*t*	*P*	*t*	*P*
WFIKKN1	0.69	0.492	−0.963	0.337	1.713	0.09	0.893	0.41	−0.401	0.69	−0.142	0.887
PLAU	0.201	0.841	0.023	0.982	−1.287	0.204	0.56	0.592	−0.385	0.701	−2.356	0.02
OASL	−1.759	0.081	−1.355	0.178	−0.929	0.357	0.116	0.913	−1.946	0.056	−1.208	0.23
FGF17	1.194	0.236	−0.53	0.597	2.541	0.013	1.81	0.1	−0.688	0.494	1.472	0.145
NPPA	0.404	0.687	−1.054	0.294	1.31	0.194	1.382	0.226	0.463	0.644	1.933	0.056
IL20RB	0.51	0.611	−1.585	0.116	−1.715	0.094	1.982	0.067	−1.894	0.064	−2.584	0.012
MET	2.01	0.047	−0.561	0.576	−3.355	0.001	0.27	0.799	−2.783	0.007	−3.222	0.002
SHC2	0.586	0.559	0.304	0.761	1.598	0.114	3.112	0.012	0.163	0.871	2.184	0.031
Risk score	0.736	0.463	−0.662	0.51	−3.006	0.004	0.106	0.919	−1.966	0.054	−3.456	7.81 × 10^–4^

### Immunocyte Infiltration

**FIGURE 9:**
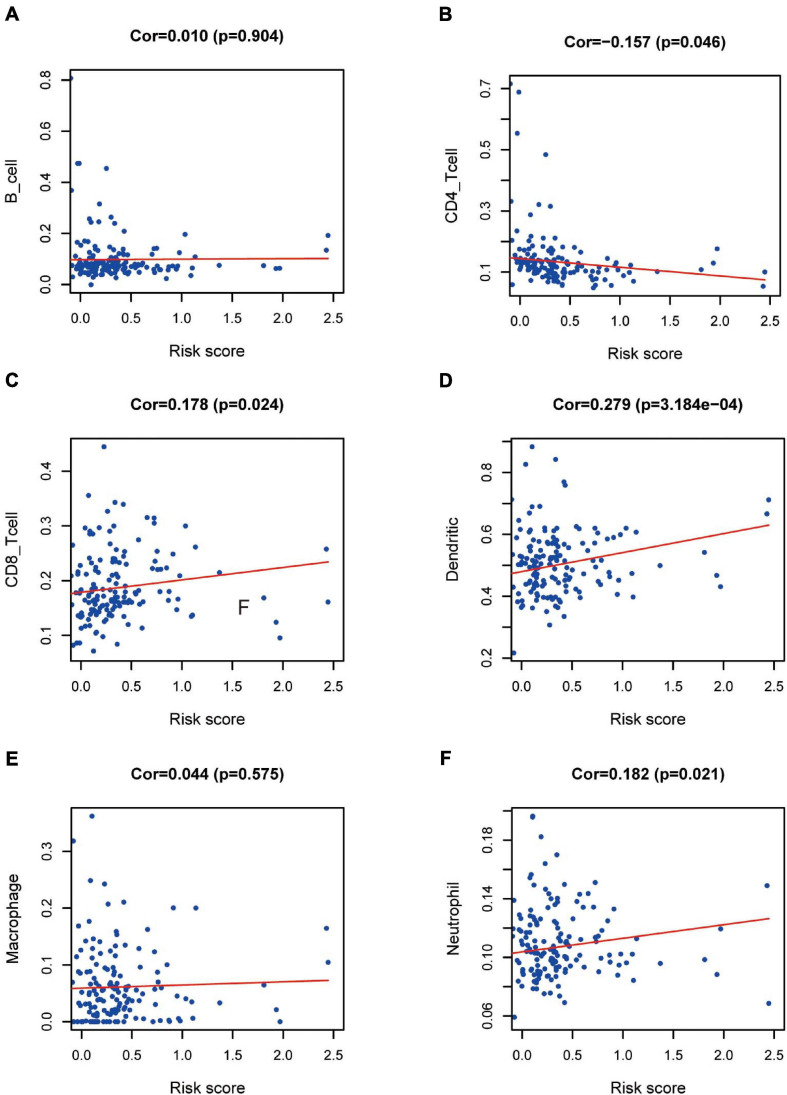
Relationships between prognostic value and degree of infiltration of six types of immune cells. The relationship of the eight-IRG prognostic model with **(A)** B cells, **(B)** CD4 T cells, **(C)** CD8 T cells, **(D)** dendritic cells, **(E)** macrophages, and **(F)** neutrophils is revealed by scatter diagrams.

We wished to ascertain if the eight-IRG prognostic model reflected the status of the PAAD immune microenvironment precisely. Hence, correlation analysis was done to explore the relationship between prognostic IRGs and infiltration of immune cells (Figure 9). The number of DCs, neutrophils, and CD8^+^ T cells was positively correlated with the risk-score prediction model (*P* < 0.05; Figures 9C,D,F) but the trend of CD4^+^ T cells was opposite (Figure 9B).

### External Verification of the Eight-IRG Prognostic Model

**FIGURE 10:**
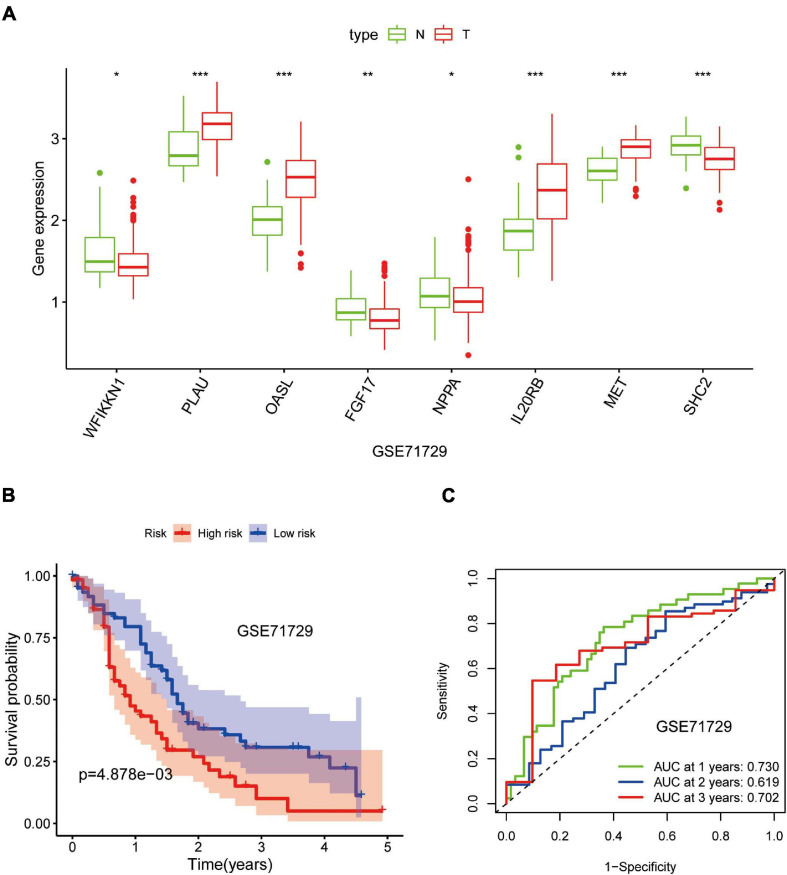
Validation of the eight-gene signature in the Gene Expression Omnibus (GEO) database. **(A)** Expression of eight IRGs in tumor tissues and normal tissues in the GSE71729 database. **(B)** Kaplan–Meier curves for low- and high-risk groups in the GSE71729 database (*P* < 0.01). **(C)** ROC curve for predicting survival from PAAD based on the risk score of the GSE71729 database. *p < 0.05; **p < 0.01; ***p < 0.001.

**FIGURE 11:**
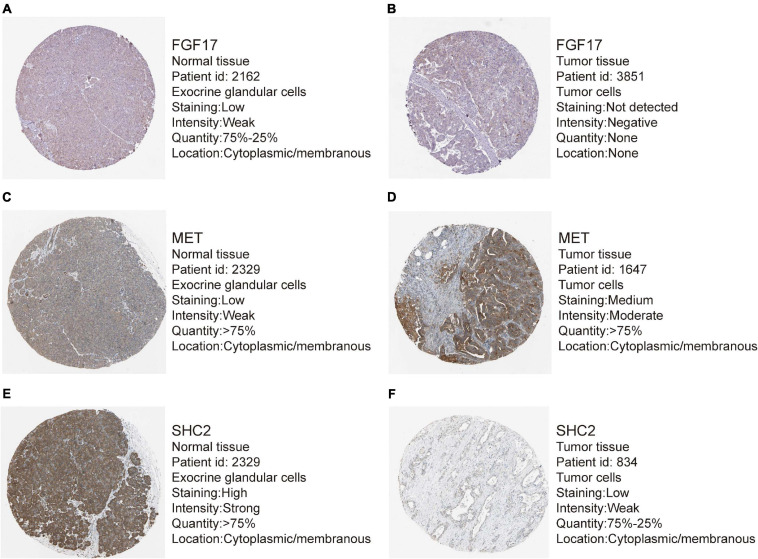
Representative immunohistochemistry images for expression of FGF17, MET, and SHC2 in pancreatic cancer tissues and normal tissues were shown with the fraction of samples with antibody staining/protein expression level high, medium, low, or not detected. **(A–B)** Expression of FGF17 in PAAD tissues were lower than that in normal tissues. **(C–D)** Expression of MET in PAAD tissues were higher than that in normal tissues. **(E–F)** Expression of SHC2 in PAAD tissues were obviously lower than that in normal tissues.

Out of the eight prognostic IRGs in our model, the expression of four IRGs was upregulated and that of the remaining four IRGs was downregulated in the TCGA–PAAD cohort. In addition, a GEO dataset (GSE71729) was used to externally verify the difference in expression of eight IRGs between tumor tissues and normal tissues. As expected, the expression of IL20RB, MET, OASL, and PLAU in tumor tissues was significantly higher than that in normal tissues. FGF17, natriuretic peptide A (NPPA), SHC2, and WAP, follistatin/kazal, immunoglobulin, kunitz, and netrin domain containing 1 (WFIKKN1) was not expressed or at a minimal level in tumor tissues (Figure 10A). The protein distribution and expression of FGF17, MET, and SHC2 are displayed in Figures 11A–F, whereas the other five IRGs remained inaccessible in the Human Protein Atlas. Verification using the GEO database further confirmed that PAAD patients in the low-risk group showed a significant OS benefit compared with that in PAAD patients in the high-risk group. The Kaplan–Meier estimator effectively distinguished different groups of various risk (*P* < 0.01; Figure 10B). The predictive capacity of the signature was confirmed by analyses of the ROC curve. Our results showed that the prognostic signatures of the GEO dataset also performed well in forecasting 1-, 2-, and 3-years survival (Figure 10C).

## Discussion

Pancreatic cancer remains a lethal type of cancer due to its poor prognosis and lack of efficacious therapeutic approaches. Precise prediction of OS after contracting PAAD is very important for the choice of therapeutic method and improving the prognosis.

Pancreatic cancer lacks reliable and effective prognostic biomarkers related to the tumor immune microenvironment. An effective prediction model to accurately assess the prognosis of PAAD is long overdue. We intended to explore DEIRGs and establish a model of PAAD based on IRGs to uncover the biomarkers that predict the diagnosis and prognosis of PAAD.

In our study, 446 DEIRGs of PC were identified by comprehensive analyses. Analyses of pathway enrichment revealed that these DEIRGs correlated with the inflammatory response and typical tumor-related pathways shown in Supplementary Table 4. Most of them were related to the progression and treatment of PAAD. Cytokines and their correlated pathways may play a relevant part in PAAD progression and immune evasion (Padoan et al., 2019; Dey et al., 2020). As a vital component of the signaling between cancer cells and surrounding stromal cells, chemokine signaling participates in the development of the supportive TME of PAAD (Sleightholm et al., 2017). The Janus kinase family/signal transducer and activator of transcription (JAK/STAT) signaling pathway were central to tumor growth, tumor survival, and systemic inflammation, particularly in PC (Quintás-Cardama and Verstovsek, 2013; von Ahrens et al., 2017). In addition, two studies (Hurwitz et al., 2015; Beatty et al., 2019) showed that inhibitors of the JAK/STAT pathway may have clinical benefit. A follow-up study of a general population indicated that the high cytotoxic activity of natural killer (NK) cells is linked to a reduced risk of cancer (Imai et al., 2000). Lee et al. (2020) stated that the activity of NK cells decreased as cancer progressed, and that decreased activity of NK cells was associated with poor clinical outcomes. NF-κB is a pro-inflammatory signaling pathway in pancreatitis and PAAD. Increased basal levels and/or inducible levels of NF-κB activation are strongly linked to several aspects of treatment resistance, as well as the proliferation and metastasis of tumor cells in PAAD (Arlt et al., 2012; Kabacaoglu et al., 2019). In addition, NF-κB-mediated chemokine signaling plays a crucial part in the therapy resistance of PC (Geismann et al., 2019). Signaling by T-helper (Th1) and Th2 cytokines is complex in the microenvironment of pancreatic tumors (Andrianifahanana et al., 2006). The presence of tumor-infiltrating lymphocytes with high Th2:Th1 ratios demonstrates a poor prognosis in PAAD (Seicean et al., 2009). He et al. (2011) revealed that the accumulation of Th17 cells and their relevant cytokine levels in PC tissues may manifest engagement in the invasion and metastasis of PC, which may thereby have an impact on the prognosis. We conducted a comprehensive investigation of the biological functions of DEIRGs in PAAD populations to provide a basis for elucidating their possible molecular regulatory mechanisms.

More and more studies have found that abnormally expressed TFs in tumor tissues were related to aggressive diseases and poor prognosis. The research on new drugs that target specific TFs had great potential in developing clinically relevant strategies for the treatment of malignant tumors (Sankpal et al., 2012). To further investigate the possible molecular regulatory mechanisms, a TFs-mediated prognosis-related IRGs network was structured to find the significant TFs regulating DEIRGs in this network. DETFs such as basic helix-loop-helix family member E40 (BHLHE40), E2F transcription factor 1 (E2F1), early growth response 2 (EGR2), FOS like 1, AP-1 transcription factor subunit (FOSL1), forkhead box M1 (FOXM1), kruppel like factor 5 (KLF5), LEF1, LMNB1, peroxisome proliferator activated receptor gamma (PPARG), PR/SET domain 1 (PRDM1), transcription factor AP-2 alpha (TFAP2A), tumor protein P63 (TP63), and VDR might regulate the DEIRGs in PAAD. BHLHE40 expression was upregulated by transforming growth factor (TGF)-β, and affected the morphology, migration, and invasion of PC cells by changing the expression of factors related to epithelial-to-mesenchymal (EMT) transition (Wu et al., 2012). E2F1-mediated overexpression of long non-coding (lnc)RNA-pancreatic cancer associated transcript 1 (PLACT1) promotes the growth of PAAD by continuously activating the NF-κB pathway and forming a positive feedback loop with IκBα in PC (Ren et al., 2020). Vallejo et al. (2017) revealed FOSL1 to be an oncogene in KRAS-driven lung cancer and PC, which partially factors through transcriptional regulation of a subset of genes involved in the mitotic machinery. Zhou et al. (2019) revealed an important epigenetic modification to FOXM1, and increased expression of FOXM1 suppressed the maturation of bone marrow−derived DCs via direct activation of Wnt5a signaling pathway and weakened the promotion of T−cell proliferation. He et al. (2018) demonstrated that KLF5 depletion in oncogenic Kras-expressing mouse PC cells reduced proliferation of tumor cells and PC progression. TP63 is a member of the p53 family and is transcribed from two promoters to produce two subtypes: TAp63 and ΔNp63 (Gonfloni et al., 2015). TP63 reprograms enhancers to drive squamous transdifferentiation in PC (Somerville et al., 2020). Sherman and collaborators discovered that the VDR is expressed in the stroma from PC cells and acts as a “master” transcriptional regulator of pancreatic stellate cells, thereby resulting in induced transcriptional reprogramming of tumor stroma in PAAD.

We innovatively established a TFs-mediated prognosis-related IRGs regulatory network in PAAD by bioinformatics analysis. This network showed that TFs regulated IRGs positively and negatively, which supplied a novel method to explore the IRGs underlying regulatory mechanisms in PAAD at the molecular level.

Eight IRGs involved in the prognostic model were considered to be potential biomarkers in PAAD. Among the eight genes, MET, OASL, SHC2, and PLAU have been well studied in PAAD compared with other IRGs. Nan and coworkers found that hepatocyte growth factor (HGF) promotes the invasion and migration of PC cells by activating the HGF/c-Met pathway (Nan et al., 2019). Besides, MET/HGF co-targeting may represent a treatment option for patients with PC (Modica et al., 2018). As a member of the OAS protein family, OASL is associated with the innate immune defense against viral infections. Glaß et al. (2020) identified OASL to be a candidate oncogenic RNA-binding protein with partially validated target potential in PC. Recently, PLAU has been reported to be an oncogene that activates EMT progression in PAAD (Zhao X. et al., 2020). SHC2 was a proverbial adaptor molecule that binds to receptor tyrosine kinases via its SH2 domain. Teodorczyk and coworkers reported that CD95L could induce SCK recruitment and activation of the phosphoinositide 3-kinase/extracellular signal-regulated kinase (PI3K/ERK) pathway by stimulating CD95 receptors and, ultimately, lead to PC cell-cycle progression (Teodorczyk et al., 2015). One review stated that high expression of IL20RB was related to poor survival, thereby suggesting its oncogenic potential in PAAD (Haider et al., 2014). FGF17 was a member of the FGF8 subfamily, which promotes the development and progression of hepatocellular carcinoma (Gauglhofer et al., 2011). In addition, FGF17 was overexpressed in human prostate cancer, and involved in the progression of prostate cancer to high−grade disease (Heer et al., 2004). Both studies have reported that FGF17 may be a novel tumor-promoting gene whose expression is upregulated in neoplasms, data which contradicted our findings. The exact role of FGF17 in PAAD is not known. Few related studies have reported NPPA or WFIKKN1 being involved in PAAD.

Our TFs-IRGs-mediated network contained five of the eight modeling genes: PLAU, OASL, FGF17, MET, and SHC2. Their interactions with tumor-associated TFs can provide a certain theoretical direction/basis for mechanistic studies. Therefore, further study of the potential regulatory mechanisms of these prognostic immune genes in PAAD is needed.

To clarify the immune microenvironment in PAAD, a correlation analysis on immunocytes was done based on the TIMER database. Results indicated that lower infiltration of DCs, CD8^+^ T cells, and neutrophils may be observed in low-risk patients, whereas the tendency of CD4^+^ T cells was the opposite. DCs, neutrophils, and CD8^+^ T cells exhibited a significantly positive regulatory relationship with the prognostic model. Thus, our model may act as a predictive factor for increased infiltration of immune cells. One study reported that higher numbers of CD4^+^ T lymphocytes were significantly associated with longer survival, which echoed our findings (Ino et al., 2013). A recent study showed that intratumoral infiltration by CD8^+^ T lymphocytes and neutrophils and a favorable prognosis in PAAD patients were tightly linked (Miksch et al., 2019), which is the reverse of our results. Thus, our results must be validated by further investigations. Whether the infiltration level of DCs in tumors indicates the clinical prognosis of PAAD patients has not been reported. Studies on other tumors have yielded inconsistent or even conflicting results that doubt the value of infiltrating DCs (Karthaus et al., 2012). The exact role of immunocytes in PAAD has not been clarified. Considering the different levels of immunocyte infiltration between high-risk and low-risk PAAD groups, suitable immunotherapy strategies can be selected based on the basis of the immune microenvironment in PAAD.

So far, several studies have proposed that prognostic gene signatures based on mRNA levels can predict the OS of PC prognosis. For instance, Birnbaum et al. (2017) built a 25-gene classifier that helped select patients with resectable disease for immediate surgery or neoadjuvant chemotherapy. Another study established a four-gene signature for prediction of OS from PC based on gene-expression data from the GEO database [1-, 2-, and 3-years survival area under the curve (AUC) reached 0.715, 0.654, and 0.715, respectively] (Yan et al., 2019). A recent study investigated the survival-associated genes from the integrated analysis of multiple datasets, and established prognostic signatures in PAAD (1-, 2-, and 3-years survival AUC reached 0.699, 0.637, and 0.621, respectively; Wu et al., 2019). At present, there are few studies on the relationship between IRGs and the prognosis of PAAD. The latest research developed an immune prognostic model to identify low-risk patients who may benefit from immunotherapy (Gu et al., 2021). However, this predictive model still lacks an external cohort to verify the effectiveness of the model. We used a specialized immune database to explore the relationship between many IRGs and the prognosis of PAAD patients. Subsequently, we established a new immune-related prognostic signature. No overlap was found between the eight-gene signatures we developed and the one defined previously. Besides, the riskscore had a robust predictive performance with 1-, 2-, and 3-years survival AUC reached 0.750, 0.697, and 0.704, respectively. The predictive performance of our prognostic model was superior or comparable with that reported in other studies, and this model prediction is verified in an external validation cohort. These results suggest that an immune-related prognostic signature may be a valid marker for the prediction of the PC prognosis.

Nevertheless, our study still has perceived limitations. Firstly, we only used data from sections of public databases to build and validate our prediction model. Therefore, one must conduct more prospective studies to verify its clinical applicability. Secondly, we excluded many prominent prognostic genes in PAAD, so the potential weakness inherent in constructing a prognostic model with a single hallmark is inevitable. Moreover, the protein expression of IRGs related to the prognosis, and their potential molecular mechanisms in the pathogenesis and development of PAAD, must be confirmed by additional experimental studies.

## Conclusion

We defined a novel eight-IRG model as an independent prognostic predictor for PAAD. The prognostic value of this model was verified by an external validation database. Moreover, the correlation between the eight-IRG prognostic model and infiltrated immunocytes could demonstrate its pivotal role in the PAAD immune microenvironment, which could be utilized as a new prognostic and therapeutic biomarker in PAAD patients.

## Data Availability Statement

The original contributions presented in the study are included in the article/Supplementary Material, further inquiries can be directed to the corresponding authors.

## Author Contributions

MM, HL, and YL wrote the main manuscript text. MM and HL prepared figures and tables. YC, BX, and RZ reviewed the manuscript. All authors contributed to the article and approved the submitted version.

## Conflict of Interest

The authors declare that the research was conducted in the absence of any commercial or financial relationships that could be construed as a potential conflict of interest.

## Footnotes

1 www.gtexportal.org/

2 https://www.ncbi.nlm.nih.gov/geo/

3 www.immport.org/

4 www.cistrome.org/

5 www.bioconductor.org/packages/release/bioc/html/limma.html

6 https://david.ncifcrf.gov/

7 https://cran.r-project.org/web/packages/GOplot/citation.html

8 www.bioconductor.org/packages/release/bioc/html/clusterProfiler.html

9 https://cran.r-project.org/web/views/Survival.html

10 https://cran.r-project.org/web/packages/glmnet/index.html

11 https://cran.r-project.org/web/packages/survivalROC/index.html

12 www.proteinatlas.org/

13 https://cistrome.shinyapps.io/timer/

14 www.cytoscape.org/
