# Supplementary material for: Construction and Validation of an Immune-Based Prognostic Model for Pancreatic Adenocarcinoma Based on Public Databases
Source: Front Genet. 2021 Jul 14;12:702102. doi: 10.3389/fgene.2021.702102 (PMC8318842; doi:10.3389/fgene.2021.702102)
Supplement: Supplementary file 2 [file Data_Sheet_2.DOCX]

Supplementary Material


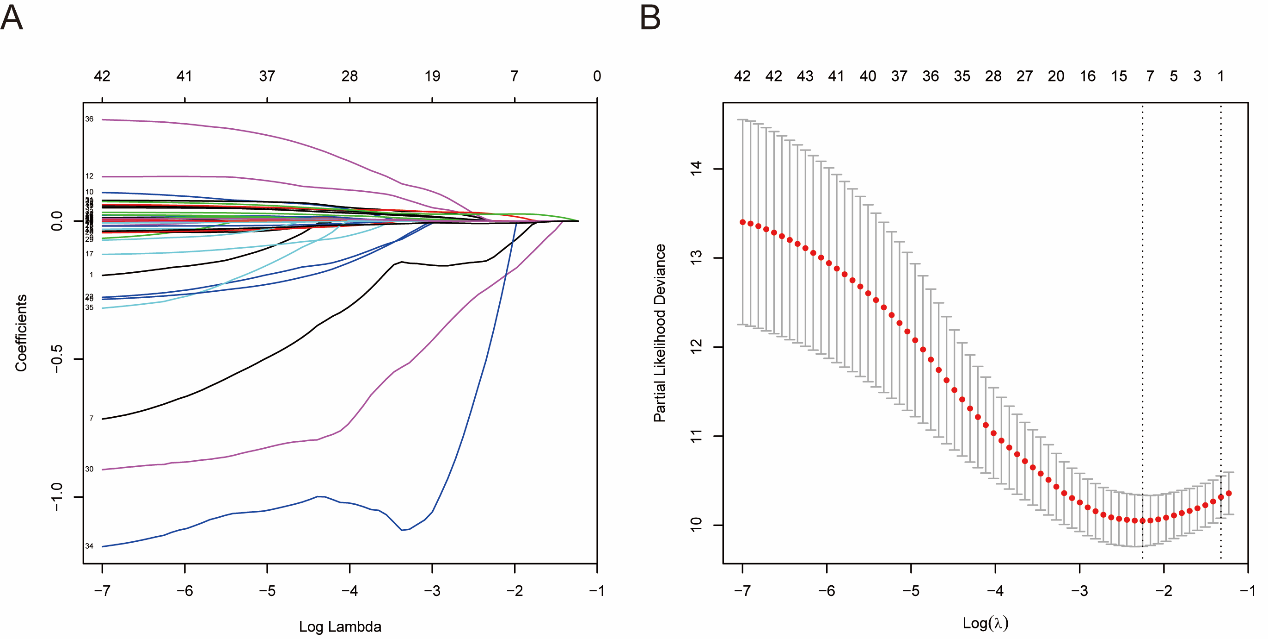


**Supplementary Figure 1.** Construction of an eight-gene signature model in the TCGA-PAAD cohort. (A). LASSO coefficient profiles of the expression of 43 candidate genes. (B). Selection of the penalty parameter (λ) in the LASSO model via 8-fold cross-validation. The dotted vertical lines are plotted at the optimal values following the minimum criteria (left) and “one standard error” criteria (right).
